# Compulsive exercise in eating disorders: proposal for a definition and a clinical assessment

**DOI:** 10.1186/s40337-018-0219-x

**Published:** 2018-11-28

**Authors:** Nina Dittmer, Corinna Jacobi, Ulrich Voderholzer

**Affiliations:** 1 Schoen Clinic Roseneck, Am Roseneck 6, Prien am Chiemsee, Germany; 2 Department of Clinical Psychology and E-Mental-Health, Technische Universität Dresden, Dresden, Germany; 3 Department of Psychiatry and Psychotherapy, University of Freiburg, Freiburg, Germany; 4 Department of Psychiatry and Psychotherapy, University of Munich (LMU), Munich, Germany

**Keywords:** Eating disorders, Compulsive exercise, Definition, Assessment, Clinical interview, Consensus

## Abstract

**Background:**

Compulsive exercise has been recognized as a highly prevalent symptom in eating disorders (ED) for over 100 years and is associated with poor short-term and long-term treatment outcome. Progress in understanding and treatment of compulsive exercise will remain limited as long as no consensus framework for definition and assessment of compulsive exercise exists, as results cannot be compared across clinical studies.

Based on existing literature, it was our aim to propose a transdiagnostic definition and a clinical assessment for compulsive exercise, that can be applied to adolescent and adult patients with ED.

**Method:**

During a series of meetings of experienced clinicians at a highly specialized hospital for eating disorders, we elaborated a transdiagnostic definition of compulsive exercise in ED. Additionally, we derived a clinical interview for the assessment of compulsive exercise and its different subtypes.

**Results:**

The core criterion when defining and assessing compulsive exercise is a pathologically increased exercise pattern characterized by 1) excessive exercise that a patient feels driven to perform in response to an obsession or according to rules that must be applied rigidly, and 2) exercise that is aimed at preventing or reducing distress or at preventing some dreaded consequence. A second necessary criterion is the physical or psychological burden caused by compulsive exercise, i.e., that it is time-consuming, significantly interferes with the patient’s daily routine, occupational functioning or social relationships or is continued despite medical injury, illness, or lack of enjoyment. Insight that compulsive exercise is excessive or unreasonable was added as an optional criterion.

Compulsive exercise manifests itself in three different subtypes: 1) vigorous exercise, 2) marked increase in daily movement, or 3) motor restlessness.

The above criteria must be met during the past 6 months, together with one of the three subtypes of compulsive exercise.

**Conclusions:**

The proposed criteria aim to foster the discussion around definition and assessment of compulsive exercise with the goal of reaching an international consensus in the near future.

Providing a consistent framework for researchers and clinicians would considerably advance understanding and treatment of compulsive exercise in ED patients.

## Plain English Summary

Compulsive exercise represents a frequent symptom in eating disorders and is associated with poor short-term and long-term treatment outcome. Progress in research on compulsive exercise will remain limited if no common framework for definition and assessment of compulsive exercise exists, as results cannot be compared across clinical studies.

Based on existing literature, it was our aim to propose a definition and clinical interview for assessing compulsive exercise in eating disorders.

A group of experienced clinicians at a highly specialized hospital for eating disorders developed a definition and a clinical interview for assessing compulsive exercise in eating disorders: The most relevant criterion when defining compulsive exercise is an inappropriately high exercise pattern, shown by 1) repetitive exercise that a patient feels driven to perform according to strict personal rules 2) exercise that is aimed at managing distress or at preventing some feared consequence.

The second criterion is the physical or psychological burden caused by compulsive exercise.

Compulsive exercise manifests itself in three different subtypes: 1) strenuous, highly intensive sports, 2) increased movement in everyday life, or 3) motor restlessness.

The above criteria must be met during the past six months, together with one of the three subtypes of compulsive exercise.

In providing a common framework for researchers and clinicians we aim to considerably advance understanding and treatment of compulsive exercise.

## Background

Compulsive exercise (CE) has been included in the very first historical descriptions of Anorexia nervosa (AN): In his classical description of AN, Gull [1] depicted the following phenomenon: *“As part of the pathological history, it is curious to note […] the persistent wish to be on the move, though the emaciation was so great and the nutritive functions at an extreme ebb”.* In several studies in the twentieth century, CE was described in patients with AN and Bulimia nervosa (BN), but was not always regarded as relevant for treatment [2–6]. This changed as different studies showed that CE in eating disorder (ED) patients is associated with longer hospital stays [7] and suicidal behavior [8] and represents a significant predictor for relapse [9] and chronic course of the disorder [10, 11]. It also turned out that CE represents the most frequent compensatory behavior in children and adolescents with ED and is regarded as “gateway behavior” for further compensatory behaviors like vomiting or use of laxatives [12]. Prevalence rates range between 31 and 81% [13–16] in patients with AN and 20–66% [17–19] in patients with BN. However, only over the past years the importance of integrating healthy exercise in ED treatment was recognized [20–22] and three comprehensive treatment approaches were developed [23–25]. As pointed out repeatedly, progress in research on CE will remain limited as long as no consensus framework for definition and assessment of CE exists, because results cannot be compared across clinical studies [26–28]. So, providing a consistent framework should considerably advance understanding and treatment of CE for researchers and clinicians. A first step forward has been reached by the Delphi Study of Noetel and colleagues [27]: CE was shown to be the preferred term for describing the phenomenon. Additionally, consensus was reached on a range of items relevant for future definition and assessment of CE. In this paper, we would like to contribute to the discussion by proposing a precise transdiagnostic definition and a clinical assessment for CE. Both should be applicable to adolescent and adult patients with AN, BN, Atypical AN and BN of low frequency and/ or limited duration (both belong to Other Specified Feeding and Eating Disorders in the DSM-5) and should be based on the existing literature and in close alignment to the Delphi Study.

## Methods

Definition and clinical assessment of CE were elaborated by a panel of senior clinicians and researchers from the fields of psychiatry, clinical psychology and exercise therapy at a highly specialized hospital for eating disorders (Schoen Clinic Roseneck, Germany). Special importance was given to reflect both recent research results and clinical observations made during the panelists‘ longstanding experience with ED patients. In addition, definition and assessment should have a transdiagnostic character with applicability for both adolescent and adult patients with AN, BN, Atypical AN, and BN of low frequency and/ or limited duration (both belong to Other Specified Feeding and Eating Disorders in the DSM-5). During a series of structured discussions focusing on the individual aspects of the phenomenon, the panelists first agreed upon a working definition and preliminary assessment of CE. During a feasibility study for a new treatment approach for CE [25] the working definition and assessment were applied, clinically tested and continuously refined. With this iterative process, the panelists finally reached consensus to define and assess CE.

## Results

## Proposal for a definition of compulsive exercise

Our proposed definition of CE is presented in Table 1.

**Table 1:** Proposed definition for compulsive exercise

Compulsive exercise	
A. Compulsive exercise as defined by (1) *and* (2): 1) Excessive exercise that the patient feels driven to perform in response to an obsession or according to rules that must be applied rigidly. 2) The exercise is aimed at preventing some dreaded consequence or at preventing or reducing distress, often based on distorted beliefs about exercise.	
B. The compulsive exercise is time-consuming (takes more than 1 hour a day), significantly interferes with the person’s daily routine, occupational functioning or social relationships or is continued despite medical injury, illness, or lack of enjoyment.	
C. At some point during the course of the disorder the patient has recognized that the compulsive exercising is excessive or unreasonable.	
*Criteria A. + B. are considered obligatory, criterion C. is optional.*	

### Criterion A - Core features: Excessive, driven, rigid exercise routine, and avoidance of feared consequences or aversive emotions

Criterion A is composed of the two core features of CE.

1. Excessive, driven, rigid exercise routine

The term “excessive” was used in numerous studies where CE was defined quantitatively as inappropriately high amount of exercise, i.e. by exceeding previously defined limits concerning frequency, intensity and/ or duration of exercise [4, 14, 15, 29–34]. To decide whether or not the amount of exercise is inappropriately high, the clinician has to consider different factors like physical condition, BMI, age, gender, energy intake [35]: Whereas training of 1.5 hr as part of a sports team may be totally fine for a healthy, normal weight young adult with regular meals, sit-ups for even 5–10 min may be life-threatening for a severely underweight, malnourished patient with medical complications. Due to its frequent use in previous studies, we decided to integrate the term “excessive” into our definition to refer to the inappropriately high amount of exercise, but would like to highlight the importance of taking into account all of the above mentioned factors rather than employing a fixed amount of exercise.

Besides the amount it seems central to add the compulsive quality to a definition of CE: A subjective feeling of the exercise being “driven”, “out of control” or “compelled” represented an integral part of a definition of CE from early studies onwards [12–14, 19, 29–37]: Patients usually describe a high urge to perform their daily exercise routine and perceive it as obligatory. They experience noticeable difficulties to control their exercise behavior even in situations where they would like to, e.g. they may have to stop several times during longer drives to go for a walk. Polivy [37] as well as Naylor and colleagues [38] additionally described obsessional beliefs in ED patients with CE.

The rigidity of the exercise behavior was also mentioned in several studies [13, 24, 35, 36, 39–42]: Patients have to strictly keep to their repetitive daily exercise routine comprising e.g. fixed sequences of exercises or certain walking rounds at fixed times. Changing or interrupting this routine leads to distress. The aspect of rigidity is also represented in the Compulsive Exercise Test (CET; [43, 44]) and the Commitment to Exercise Scale (CES; [45, 46]), which belong to the most frequently used questionnaires for assessing CE in ED: The CET contains the subscale “exercise rigidity”, the CES the subscale “obligatory aspects” of exercise.

2. Avoidance of feared consequences or aversive emotions

Most patients report intense concerns about possible negative consequences of reducing or stopping their daily exercise routine. Originally, it was assumed that these feared consequences center around weight gain and that CE serves purely as “inappropriate compensatory behaviour in order to prevent weight gain” [47, 48]. Earlier studies often described a direct relationship between caloric intake and the following amount of exercise exists, which was labelled “debting” [42]. Some patients also exercise not only to “make up” for meals by “burning up” the ingested calories afterwards, but rather exercise in advance “to earn their meal” [42]. “Weight and shape control” as a more encompassing motivational factor for CE was depicted in several studies [8, 13, 39, 40, 49–51]. This aspect is also referred to in the respective section for assessment of CE in the Eating Disorder Examination (“intense exercising to control shape or weight”) [52, 53].

Newer research indicated that CE is not only maintained for weight and shape control. Over the past years, negative affect regulation has been established as a separate factor maintaining for CE [31, 39]: Most patients report intense feelings of “guilt”, “depression”, “anxiety”, “distress” or “irritability” when exercise is missed [13, 17, 39, 46, 51, 54, 55]. It is assumed that in these patients CE is maintained by negative reinforcement, i.e. by alleviating or preventing these aversive emotions.

ED patients with perfectionistic personality traits [17, 30] often cite loosing self-control, not reaching a desired feeling of achievement, or not being special anymore (e.g. “I loose control over myself.”, “I would have to recognize that I am not able to achieve anything.”, or “I would be average, exercising is the only area where my performance really sticks out.”) as main motivation for CE.

Given this variety of possible feared consequences and aversive feelings, we chose the more encompassing description “exercise is aimed at preventing some dreaded consequence or at preventing or reducing distress”.

A lot of patients hold dysfunctional, unrealistic beliefs about exercise itself or the extent of feared consequences if not exercising, e.g. “If I sit or lay down, my muscles will immediately turn into fat and I will look flabby and fat. By exercise the reverse will happen.”, “If I do not exercise, I am lazy, feckless and worthless.”, or “Exercise is less harmful than vomiting.”. So we added the phrase “often based on distorted beliefs about exercise”.

### Criterion B: Negative impact on life

Apart from the presence of these two core features, at least one of following signs of physical or psychological burden should also be present in ED patients with CE: All of them show different aspects of how the exercise behavior started having a stressful or even harmful impact on the patient’s life.

- The exercise interferes with the person’s daily routine, occupational functioning or social relationships:

A lot of our adult patients described that they had to start exercising very early in the morning to have “fulfilled their exercise workload” before waking up their children. Otherwise they would not have been able to bear sitting for several hours at their office job. Adolescent patients frequently had difficulties completing their homework due to their rigid exercise routine in the afternoon. Other patients reported about spending all of their leisure time in the gym thus jeopardizing their partnerships and friendships. This interference with usual life was also regularly described in previous studies [13, 17, 37, 56–58].

- The exercise is time-consuming:

Whether and how to define a quantitative threshold for daily or weekly exercise remains subject to discussion: Shroff and colleagues [17] defined 3 hr a day as cut-off point for excessive exercise. Davis, Kennedy and colleagues [59] and Penas-Lledo and colleagues [50] used a minimum of 5 hr per week/ five times per week for at least 1 hr. In another series of studies the cut-off was set at a minimum of 6 hr per week/ six times per week for at least 1 hr [14, 30, 33, 34, 49, 60, 61].

Similar to Brewerton and colleagues [29] and Favaro and colleagues [15], we agreed upon more than 1 hr a day as cut-off criterion: For us, it was important to reflect the obligatory nature of the exercise behavior by using a cut-off concerning daily instead of weekly exercise. Concerning a minimum of 1 hr as cut-off we aligned to the WHO-“recommendations on physical activity for health” [62]: For adolescents, up to 1 hr of physical activity per day was recommended, for adults 150 min per week. As this recommendation was made for healthy adolescents and adults, exceeding 1 hr per day will most likely be harmful, given that our patients suffer from a serious psychiatric disorder with physical consequences.

- The exercise is continued despite medical injury or illness:

A characteristic of ED patients is to continue exercise despite medical injury or illness [17, 42, 47, 56, 58, 63]. In several of our patients, stress fractures, common in AN patients with CE, exacerbated if exercise was not checked. Analogue case reports can be found in the literature [42].

- The exercise is continued despite lack of enjoyment:

A considerable amount of patients experiences their daily exercise routine as a chore where they do not derive enjoyment from anymore [19, 64]. Especially patients who did not engage in regular sports before their ED, regularly describe to actually “hate” their daily exercise routine. This aspect is also reflected in the subscale “Lack of exercise enjoyment” of the CET [43].

### Criterion C - optional: Insight, or motivation to change

We chose to include insight as optional criterion only due to the following reason:

In some studies, patients were asked whether their exercise had been “excessive” [14, 30, 34, 60]. In our experience, however, patients may very well be able to describe an excessive, rule-driven exercise routine (Criterion A) and to identify, in which ways CE negatively impacts their life (Criterion B), but will not judge it as “excessive” or “unreasonable” when directly asked. So, we included insight as optional criterion: We believe that it will give a clinician relevant information about the present level of insight into and/ or distress about CE and the resulting level of motivation of change.

## Proposal for a clinical assessment of compulsive exercise

For clinical assessment of CE, we consider four elements decisive, that we would like to outline here: First, we will present a clinical interview for assessment of CE that we derived from the proposed definition of CE. Second, we will describe the assessment of three different subtypes of CE. Third, we would like to explain the applied time frame of 6 months concerning assessment of CE. Last, we will present the applicability of the proposed clinical assessment.

### Clinical interview for assessment of CE

For clinical applicability, we translated our definition into the following clinical interview for the assessment of CE in patients with ED (Table 2):

**Table 2:** Clinical interview for assessment of compulsive exercise

Element of definition	Clinical interview question
A1. Excessive exercise that the person feels driven to perform in response to an obsession or according to rules that must be applied rigidly.	Do you have to exercise over and over again and can’t resist doing so? For clarification (if needed): Do you feel bad if you cannot exercise as you usually do? Is there a fixed exercising routine that you have to follow?
A2. The exercise is aimed at preventing some dreaded consequence or at preventing or reducing distress, often based on distorted beliefs about exercise	Why do you have to exercise? What would happen if you did not exercise?
B. The compulsive exercise is time-consuming (takes more than 1 hour a day), significantly interferes with the person’s daily routine, occupational functioning or social relationships or is continued despite medical injury, illness, or lack of enjoyment.	How much time do you spend exercising? What effect does your exercise behavior have on your life? Do you continue exercising when sick or injured? Does your exercise behavior bother you a lot?
C. At some point during the course of the disorder the person has recognized that the compulsive exercising is excessive or unreasonable.	Do you exercise more than you should or that makes sense?

For evaluation of the answers, the following algorithm applies: If the answers to questions A1, A2 and at least one of the four questions of B indicate CE, the subtype of CE is further specified.

### Assessment of three different subtypes of CE

We decided to differentiate between three subtypes of CE that were described in previous studies: 1. vigorous exercise [11, 31, 41], 2. marked increase in daily movement [41, 42], and 3. motor restlessness [41]. We are aware that in acute AN, motor restlessness represents an involuntary, starvation-dependant phenomenon mediated by neurobiological factors [65–69]. However, as a lot of weight-recovered AN as well as normal weight BN patients in our hospital show motor restlessness as well, we decided to include it nevertheless. In our clinical experience, vigorous exercise and marked in increase in daily movement as well as marked increase in daily movement and motor restlessness frequently co-occur. Severely ill patients may exhibit all three subtypes.

For assessment of the subtype of CE, we used the following clinical interview (Table 3):

**Table 3:** Subtypes of compulsive exercise

Subtype	Clinical interview question
Vigorous exercise	During the past 6 months, have you consciously ensured to e.g. regularly run, bike, skate, swim or perform strenuous exercises such as sit-ups or push-ups, despite being underweight or physically weak? Important: Please exclude school sports from your considerations! If yes, which forms of exercising did you carry out? On how many days per week? How many hours per day/week did you spend exercising? Which rules did exist regarding your daily resp. weekly exercise routine?
Marked increase in daily movement	During the past 6 months, have you consciously ensured to e.g. bike to work instead of taking the bus, to take the stairs instead of the elevator, to carry out strenuous housework, to take long walks independent of the weather, or to carry out activities standing instead of sitting? If yes, how did you incease your daily movement? On how many days per week? How many hours per day/week did you consciously increase your daily movement? Which rules did exist regarding your daily movement?
Motor restlessness	During the past 6 months, did you struggle to sit still without e.g. moving your arms, fidgeting with your legs or tensing your abdominal or leg muscles? If yes, in which form did your restlessness appear? On how many days per week? How many hours per day/week were you restless? Which rules did exist regarding your restlessness?

The order of the clinical interview for assessment of CE and of the assessment of the three different subtypes of CE is changeable. However, we would like to highlight at this point, that we do not consider it enough to only establish whether or not one of these different exercise subtypes is present. We regard it as paramount to assess whether the qualitative criteria of CE as outlined in our clinical interview are met. If only assessing whether e.g. vigourous exercise is present without asking for the compulsive quality of the exercise behavior, each competitive athlete would errorenously be classified as suffering from CE.

### Proposed time frame of assessment

We would like to also shortly discuss the applied time frame of 6 months concerning persistence of CE:

In previous studies concerning CE, time frames of 1 [11, 13, 14, 31, 34, 51, 60] or 3 months [49, 53, 61] were used. For less severely ill patients in an outpatient setting it may be sufficient to track exercise behavior over this time frame.

We chose a time frame of 6 months as we considered it relevant to track the course of CE over several months due to the following reason: Patients admitted to inpatient treatment in our hospital often suffered from a severe exacerbation of their symptomatology in the weeks/ months before admission so that they were forced to completely cessate exercise. They simply got too physically weak to exercise and/ or they were strictly forbidden to exercise by significant others or by their responsible physician. So, exercise behavior 1 to 3 months preceding hospital admission may not represent their “usual” CE routine.

### Applicability of the proposed clinical interview

The proposed clinical interview can be applied in different treatment contexts, i.e., in inpatient and outpatient settings. We purposely did not include measures like accelerometry in our assessment, as they may not be available in some settings.

We encourage clinicians to routinely assess and address CE in all ED patients: In children and adolescent patients with ED, CE has been shown to be a “gateway behavior” [12] to other compensatory behaviors. CE has also been identified as significant predictor for relapse and chronic course of the disorder [7–11]. So it seems crucial to detect and address CE as soon as possible to avoid further exacerbation of the ED and/ or poor long term outcome. The proposed clinical assessment requires little time, yet provides useful insights: It can be administered within 5-10 min and gathers relevant information concerning maintaining factors of CE and the negative impact on a patient’s life. This information may help increase motivation for change concerning CE and may provide a starting point regarding the identification of relevant underlying factors of CE.

## Discussion

The aim of this paper was to propose a transdiagsnotic definition and a clinical interview for assessment of CE that can be applied to adolescent and adult patients with ED. In the discussion, we would like to cover three additional aspects: First, we would like to comment on possible differential diagnoses. Second, we would like to compare the proposed definition with the results of the Delphi Study. Third, we would like to highlight strengths and limitations of our paper.

### Differential diagnoses

We consider the differential diagnosis between CE in ED patients, and restlessness in patients suffering from ED and comorbid Attention deficit hyperactivity disorder (ADHD) relevant. We consider it crucial to exactly assess exercise patterns: While increased exercise associated with ADHD will occur and is even recommended e.g. around longer periods of concentrated work to increase cognitive functioning [70], CE will often take place around mealtimes or around emotionally stressful situations. Furthermore, patients exercising due to ADHD do also not exhibit a compulsive, rigid daily exercise routine.

It may also be challenging for clinicians to differentiate between CE in ED patients and restlessness in patients suffering from ED and a comorbid depressive episode with mixed features: We recommend to explore whether other symptoms like inflated self-esteem, increased talkativeness, or racing thoughts are present [71]. Additionally, patients suffering from psychomotor restlessness due to a depressive episode with mixed features will neither show a certain exercise pattern nor describe a specific ED-related motivation for their restlessness.

### Comparison of the proposed definition and the results of the Delphi-study

The Delphi study published by Noetel and colleagues [35] presents a state-of-the-art synthesis on items necessary to define CE in patients with AN. When comparing our suggested definition of CE with the results of the Delphi-Study, there is a striking overlap: Both underline the excessive, driven and rigid nature of exercising, the distress caused by the inability to exercise, the continuation despite physical injury or illness and the interference with other aspects of patients’ daily routine.

Yet our definition differs from the Delphi study in three relevant criteria:

1. Delphi study: The exercise is used to compensate for caloric intake.

Our view: As outlined above, recent research as well as our own clinical experience showed a larger variety of motivations for CE in ED patients, e.g. the fear to lose control of weight and shape, the anxiety to be overwhelmed by aversive emotions, or the concern to lack a sense of achievement without exercising. Therefore, we decided to use the broader term “preventing or reducing distress or preventing some dreaded consequence” when describing the underlying motivation for CE.

2. Delphi study: The exercise is performed surreptitiously or in secret.

Our view: We agree that secrecy is an important characteristic of patients with ED and CE. It was crucial for the panelists, however, to only include criteria that can be reliably asked for or observed in a clinical setting: First, it lies in the very nature of secret exercising that it can hardly be observed. Second, due to high ambivalence towards treatment - especially towards weight gain - and recovery, neglecting or triviliazing compensatory symptoms are characteristica of ED patients which were widely discussed and which were described for CE as well [57]. Bratland-Sanda and colleagues [72] showed, that self-reported amount of exercise was significantly lower than the objectively assessed amount of exercise. As these reasons imply that the reliability of answers when directly asking about secret exercising is questionable, we decided to not include it in our definition.

3. Delphi study: The patient spends excessive time thinking about exercise.

Our view: CE and other symptoms of an ED like food restriction or vomiting are closely interrelated. So, focusing on the time spent thinking exclusively about exercising seemed artificial from a clinical perspective to our panelists. This is why we decided to not include the parameter “excessive thinking about exercise” in our definition of CE. We rather assess the time spent contemplating about the ED symptomatology in general during the routine clinical interview at patients’ admission to our hospital.

### Strengths and limitations

Our proposal shows the following strengths: First, we considered both clinical and research evidence for the development of our definition and clinical assessment of CE. Second, our definition and clinical assessment can be used broadly: We suggest a transdiagnostic definition and a short clinical interview that may be applied in different treatment settings to adolescent and adult patients with AN, Atypical AN, BN as well as BN with low frequency and/ or intensity.

There are the following limitations to our study: First, 1000 ED patients are treated per year in our hospital and all participating researchers and clinicians have worked in ED research and treatment between 5 and 20 years. Nevertheless, it represents a limitation of this study that we developed the proposed definition and assessment of CE with clinicians from one hospital only. A multicentre approach would have strengthened the methodology. Second, our definition of „excessive" that takes into account a person’s physical condition and energy intake, introduces a greater degree of subjectivity into the assessment process. This warrants to test the inter-rater reliability of the interview.

## Conclusions

CE has already been described in earliest reports of EDs. Nevertheless, up to now, definition and clinical assessment of CE are still controversially discussed among researchers and clinicians. A consensus framework of CE is urgently required as a common base for further progress in understanding and especially treatment of CE: A shared conceptualization will facilitate the development of targeted, structured treatment approaches for CE. Zipfel and colleagues [73] recognized efficacious treatment of CE as one of the key unmet challenges in the treatment of ED. For evaluation of the efficacy of new treatment approaches, a unified assessment of CE is considered fundamental to facilitate comparability of the results across clinical studies. For clinicians, a short, but theoretically sound clinical interview of CE and its subtypes will help to establish assessment of CE as part of the routine clinical interview for ED patients. To the best of the authors‘ knowledge, this is the first paper to synthesize literature on CE to develop a definition and to systematically derive a clinical interview that assesses CE and its different subtypes. As core features we propose an excessive, driven, rigid exercise routine and avoidance of feared consequences or aversive emotions. As additional criterion we propose a negative impact of CE on the patient’s life. The proposed definition and assessment should contribute to the development of a common conceptualization of CE in the near future to ultimately advance management of CE. Successful treatment of CE will represent a milestone for overall optimization of ED treatment.

## Abbreviations

ADHD: Attention deficit hyperactivity disorder
AN: Anorexia nervosa
BN: Bulimia nervosa
CE: Compulsive exercise
CES: Commitment to Exercise Scale
CET: Compulsive Exercise Test
ED: Eating disorders
